# α-Glucosidase inhibitors from a mangrove associated fungus, *Zasmidium* sp. strain EM5-10

**DOI:** 10.1186/s13065-019-0540-8

**Published:** 2019-02-07

**Authors:** Dioxelis Lopéz, Lilia Cherigo, Luis C. Mejia, Marco A. Loza-Mejía, Sergio Martínez-Luis

**Affiliations:** 1 Centro de Biodiversidad y Descubrimiento de Drogas, Instituto de Investigaciones Científicas y Servicios de Alta Tecnología (INDICASAT AIP), Edificio 208, Ciudad del Saber, Apartado, 0843-01103 Panama City, Panama; 2 Department of Biotechnology, Acharya Nagarjuna University, Nagarjuna Nagar, Guntur, 522510 India; 3 Departamento de Química Orgánica, Escuela De Química, Facultad de Ciencias Exactas y Tecnología, Universidad de Panamá, P.O. Box 3366, Panama City, Panama; 4 Facultad de Ciencias Químicas, Universidad La Salle, Benjamín Franklin 45, Cuauhtémoc, 06140 Mexico City, Mexico

**Keywords:** α-Glucosidase, *Laguncularia racemosa*, *Zasmidium*, Triglycerides, Mangroves, Diabetes mellitus

## Abstract

**Background:**

Mangroves plants and their endophytes represent a natural source of novel and bioactive compounds. In our ongoing research on mangrove endophytes from the Panamanian Pacific Coast, we have identified several bioactive endophytic fungi. From these organisms, an isolate belonging to the genus *Zasmidium* (Mycosphaerellaceae) showed 91.3% of inhibition against α-glucosidase enzyme in vitro.

**Results:**

*Zasmidium* sp. strain EM5-10 was isolated from mature leaves of *Laguncularia racemosa*, and its crude extract showed good inhibition against α-glucosidase enzyme (91.3% of inhibition). Bioassay-guided fractionation of the crude extract led to obtaining two active fractions: L (tripalmitin) and M (Fungal Tryglicerides Mixture). Tripalmitin (3.75 µM) showed better inhibitory activity than acarbose (positive control, IC_50_ 217.71 µM). Kinetic analysis established that tripalmitin acted as a mixed inhibitor. Molecular docking and molecular dynamics simulations predicted that tripalmitin binds at the same site as acarbose and also to an allosteric site in the human intestinal α-glucosidase (PDB: 3TOP).

**Conclusions:**

*Zasmidium* sp. strain EM5-10 represents a new source of bioactive substances that could possess beneficial properties for human health.

## Background

Mangrove plants and their endophytes represent a natural source of novel and bioactive compounds that have been attracting the attention of scientists in the past decade. Mangroves are a group of 73 species of shrubs or trees found in tropical and subtropical areas. Mangroves are highly adapted to tolerate extreme conditions such as high levels of salinity, high temperature, and moisture [1]. In several parts of the world, mangroves have been used for traditional medicine. Chemical studies of mangrove species have led to the identification of more of 200 bioactive compounds [2–4]. Endophytic fungi of mangroves have been shown to be a good source of novel, bioactive, and exceptional compounds with unique and unusual structures. So far, more than 322 fungal metabolites, isolated from mangrove associated fungi, have been obtained and have showed promising biological activities [5, 6].

Hyperglycemia is a rapid increase in blood glucose levels due to starch hydrolysis by α-amylase and glucose releasing into the small intestine by α-glucosidase action. Inhibition of both enzymes should result in postprandial hyperglycemia decline, which could be an important strategy for the control of diabetes mellitus. Unfortunately, available therapeutic α-glucosidase inhibitors have a strong α-amylase inhibitory activity which can lead to digestive tract disorders such as abdominal distension, flatulence, meteorism, and diarrhea [7]. Thus, new inhibitors are required, especially ones with low α-amylase inhibition activity, because they could represent an effective therapy for postprandial hyperglycemia with minimal side effects. The first inhibitor of α-glucosidase introduced in the market was acarbose, a metabolite discovered in microorganisms from the *Actinoplanes* genus. In recent years, several fungal compounds, including those from mangrove endophytes, have been reported as inhibitors of α-glucosidase [8–13], which is evidence that these kinds of microorganisms are prolific producers of α-glucosidase inhibitors.

Recently, our lab group decided to establish a research line aimed at the systematic biological evaluation of extracts from mangrove organisms to find them potential biomedical applications [14]. In our ongoing research on mangrove plant species and their endophytes from the Panamanian Pacific Coast, several bioactive endophytic fungi have been identified. From these fungi, an isolate belonging to the genus *Zasmidium* (Mycosphaerellaceae) showed good activity against α-glucosidase enzyme in vitro. Its organic extract inhibited 91.3% of the enzyme function. Bioassay-guided fractionation allowed us to obtain two active fractions, one of which was composed by tripalmitin and the other for a triglyceride mixture. Here, we report some results obtained in this study.

## Results

### Fungal isolation and characterization

An endophytic fungus, isolate EM5-10, was obtained from mature leaves of *Laguncularia racemosa* (Combretaceae), collected from Mangroves and wetlands located in an area of the Bay of Panama known as Juan Diaz, Panama. This isolate was identified as *Zasmidium* sp., based on 99% DNA sequence identity of the ITS region of this isolate with that from the holotype of *Stenella musae* (culture CBS 122477, Accession Number EU514291.1), now under the genus Zasmidium [15]. The isolate is here identified as *Zasmidium* sp. strain EM5-10 with ITS sequence labeled as Genbank Accession Number KX898455. Further systematic work is required for accurate phylogenetic relationships of this isolate with congeneric species and for assessing the generality of the bioactive activity described in this work. In our view, this is the first report of the isolation of a species belonging to Zasmidium genus as endophytic fungi of *L. racemosa* leaves, and this finding allows us to determine that this species can tolerate a relatively high percentage of salt in its culture conditions.

### Chemical study

**Fig. 1:**
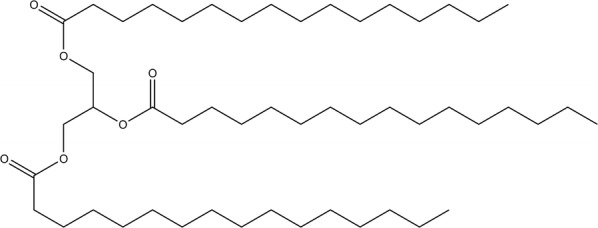
Compound 1 (tripalmitin)

**Fig. 2:**
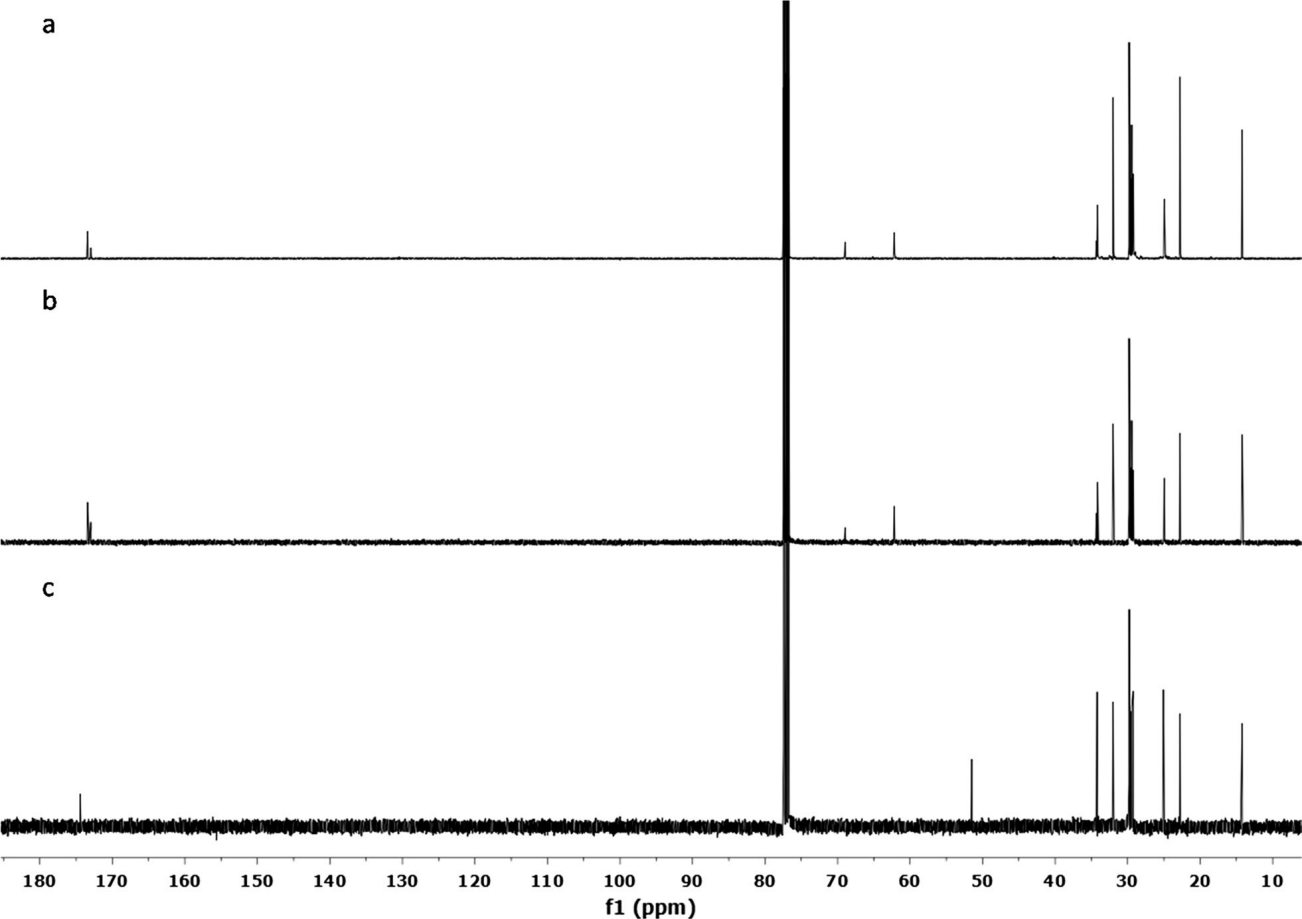
^13^C NMR Spectra (100 MHz). (a) Tripalmitin, (b) tripalmitin standard, (c) methyl palmitate (obtained from the methanolysis reaction)

In the initial screening, the crude extract showed good inhibition against α-glucosidase enzyme (91.3% of inhibition). Following the protocols of our laboratory, we performed a primary fractionation by Solid-Phase Extraction to obtain 16 fractions. All 16 fractions were submitted for bioactivity testing. Only two fractions, L and M, exhibited 97% and 96% of α-glucosidase inhibition, respectively, at concentrations of 6.25 µg/mL. Through spectroscopic analysis, we detected that both fractions had compounds of triglycerides type. Additionally, Fraction L contained one major component with approximately 97% of purity (compound **1**), and Fraction M consisted of a mixture of triglycerides (with at least two main components). Comparison of the obtained NMR data with those of the literature allow the identification of the compound as tripalmitin (Fig. 1) [16, 17]. Additionally, chemical shifts of the isolated compound were compared with those of authentic sample of tripalmitin obtained from Sigma-Aldrich, and the NMR spectra of both samples showed complete concordance (Fig. 2). In order to corroborate the presence of triglycerides, we proceeded to perform a methanolysis reaction to release the fatty acid methyl esters (FAME). The FAME formed after methanolysis were extracted and analyzed by NMR and TLC. Analysis of the results of these tests revealed that the methyl ester of palmitic acid was the main component of the reaction mixture (Fig. 2).

**Fig. 3:**
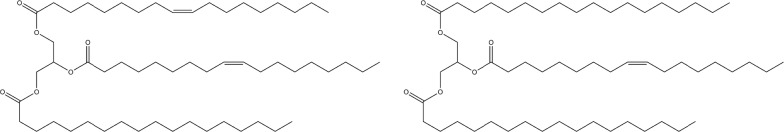
Fungal triglyceride mixture

On the other hand, active fraction M was a triglycerides mixture (FTGm), and this mixture presented an inherent difficulty for the separation of its constituents, due to this, we were unable to separate the compounds with the equipment available to us. Hence, we proceeded to identify some of its major components with the acquired spectra. The mass spectrum of fraction M exhibited two peaks sticking out over the rest of the components, with pseudomolecular ions at *m/z* 889.8211 and at *m/z* 887.8057. The molecular formula of these ions were C_57_H_109_O_6_ and C_57_H_107_O_6_, which together with NMR data analysis allows us to infer that both compounds are triglycerides containing oleic acid and stearic acid in their structure. For molecular Docking study, a hypothetical fungal triglyceride (FTG) structure was proposed which contained one chain of oleic acid located in C-2 and two chains of stearic acid in C-1 and C-3 (Fig. 3).

### α-Glucosidase inhibition evaluation and kinetic studies

**Fig. 4:**
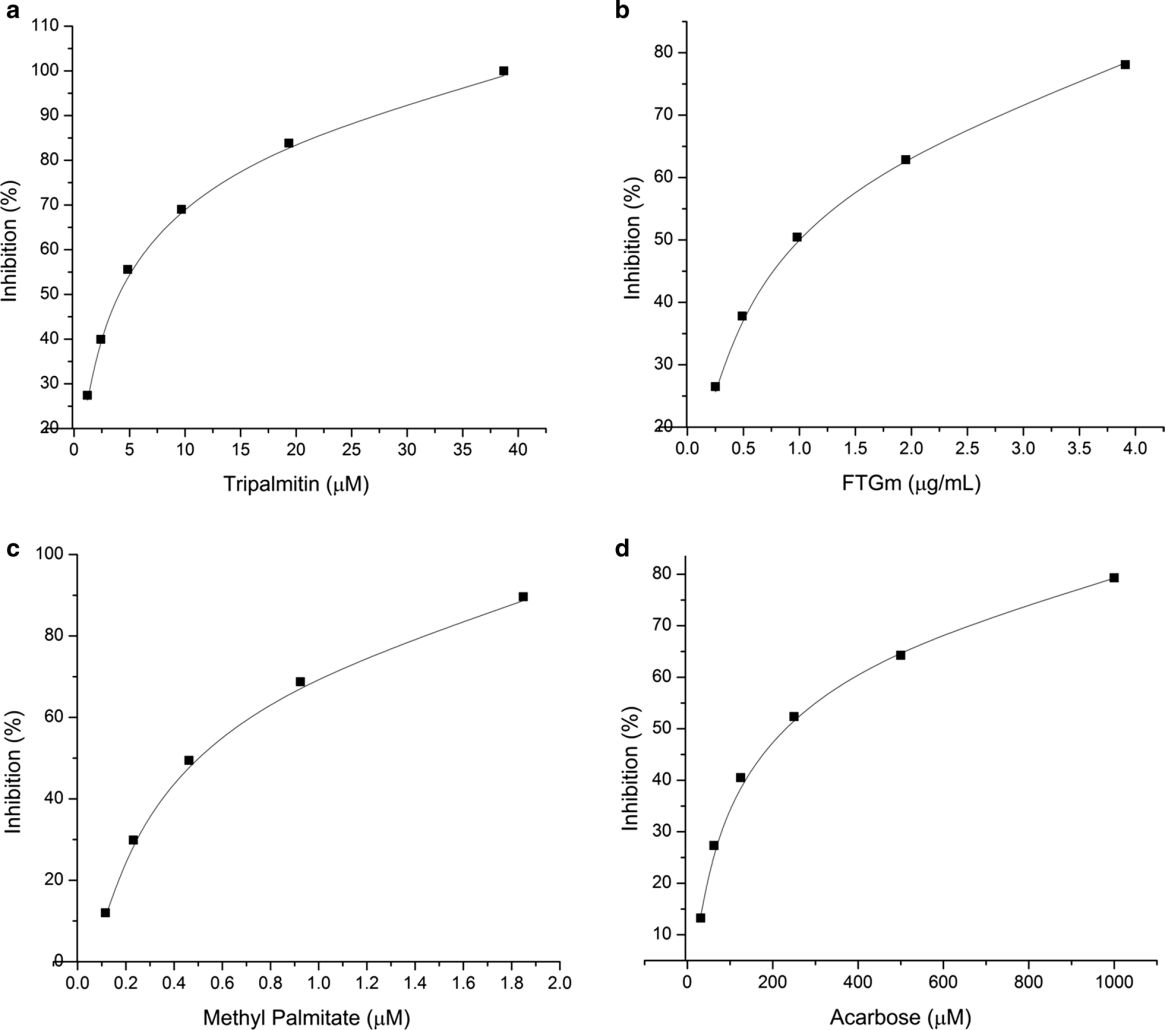
α-Glucosidase inhibition **a** tripalmitin, **b** fungal triglycerides mixture, **c** methyl palmitate, **d** acarbose

**Fig. 5:**
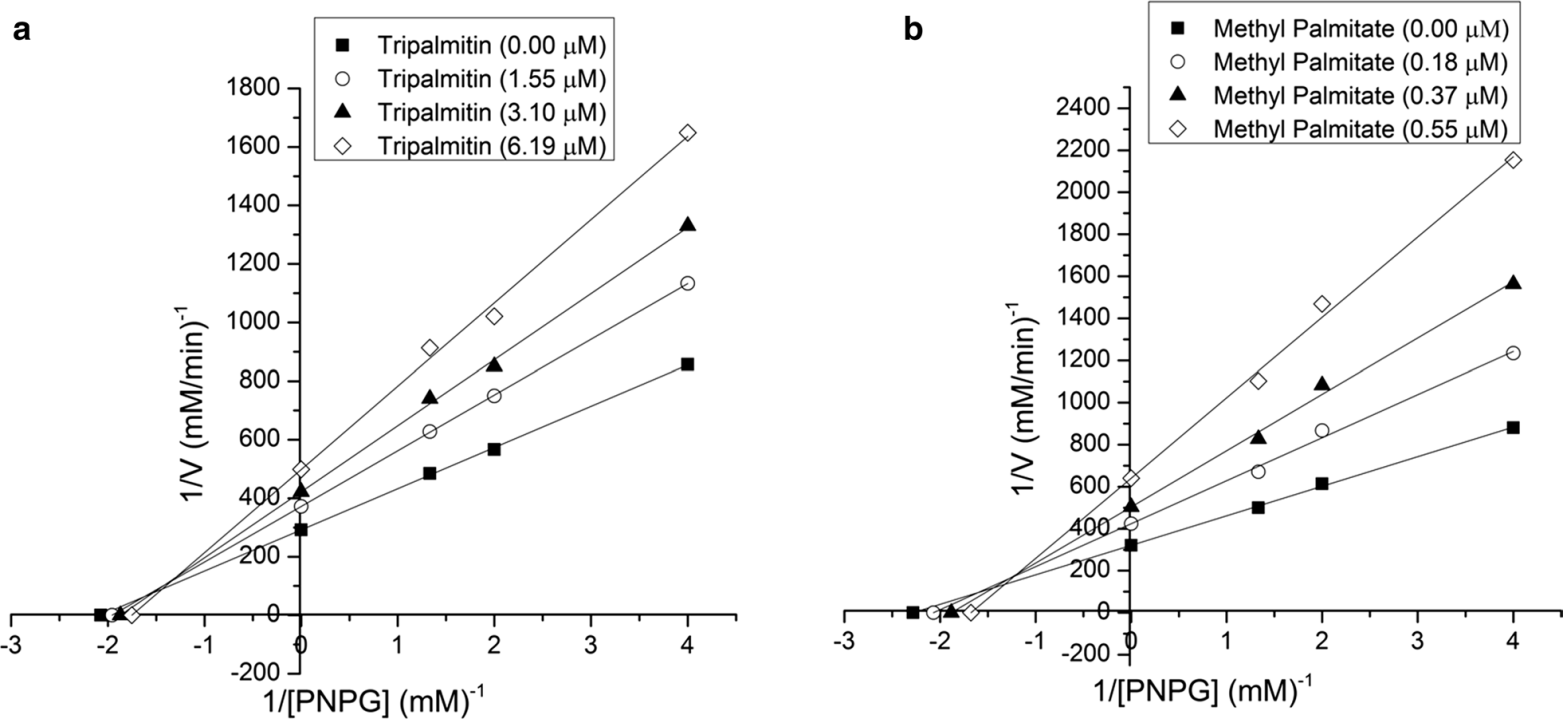
Lineweaver–Burk plots of α-glucosidase inhibitors **a** tripalmitin, **b** methyl palmitate

Tripalmitin inhibited α-glucosidase enzyme in a concentration-dependent manner with an IC_50_ value of 3.02 µg/mL (3.75 µM, Fig. 4a). On the other hand, FTG from fraction M inhibited α-glucosidase enzyme in a concentration-dependent manner with an IC_50_ value of 0.92 µg/mL (Fig. 4b). Both fractions showed better inhibitory activity than acarbose (positive control, IC_50_ 217.71 µM/140.55 µg/mL) (Fig. 4d). Kinetic analysis was carried out to understand the interaction of tripalmitin with α-glucosidase. Lineweaver–Burk plots were constructed using different concentrations of substrate and tripalmitin. Lineweaver–Burk plots in Fig. 5a demonstrated tripalmitin acts as a mixed inhibitor against α-glucosidase enzyme. Also, methyl palmitate inhibited α-glucosidase enzyme in a concentration-dependent manner (Fig. 4c) with an IC_50_ value of 0.13 µg/mL (0.46 µM). Lineweaver–Burks plots in Fig. 5b showed that methyl palmitate acts as mixed inhibitors against α-glucosidase. The kinetic parameters of α-glucosidase inhibition by tripalmin and methyl palmitate are in Table 1.

**Table 1:** Kinetic parameters of α-glucosidase inhibition by tripalmitin and methyl palmitate

Samples	K_m_ (mmol/L)	V_max_ (mmol/(L min)	*K*i (mM)
α-Glucosidase	0.365 ± 0.001	0.0041 ± 0.0001	n/a
Tripalmitin	0.511 ± 0.003	0.0027 ± 0.0001	332 ± 39
Methyl palmitate	0.483 ± 0.002	0.0024 ± 0.0002	34 ± 2

*K*_*m*_ Michaelis–Menten constant, *V*_*max*_ maximum reaction velocity, *Ki* inhibitory constant, *n/a* not applicable

### Docking study

**Fig. 6:**
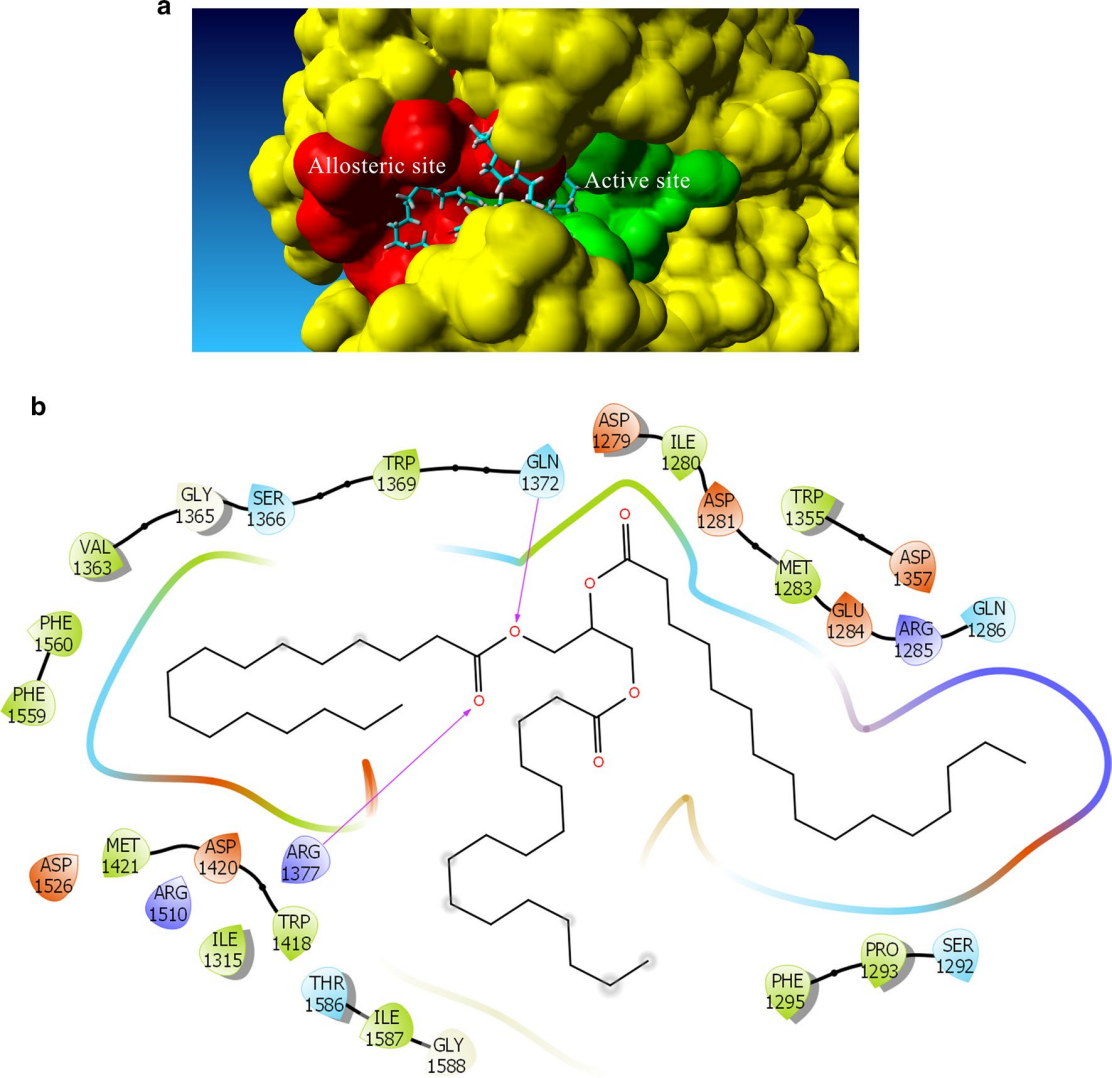
**a** Predicted pose of tripalmitin bound to the active and allosteric site of glucosidase. **b** Interaction diagram of the same binding pose. Allosteric site is comprised between Tyr 1251 to Ser 1292 sites

**Fig. 7:**
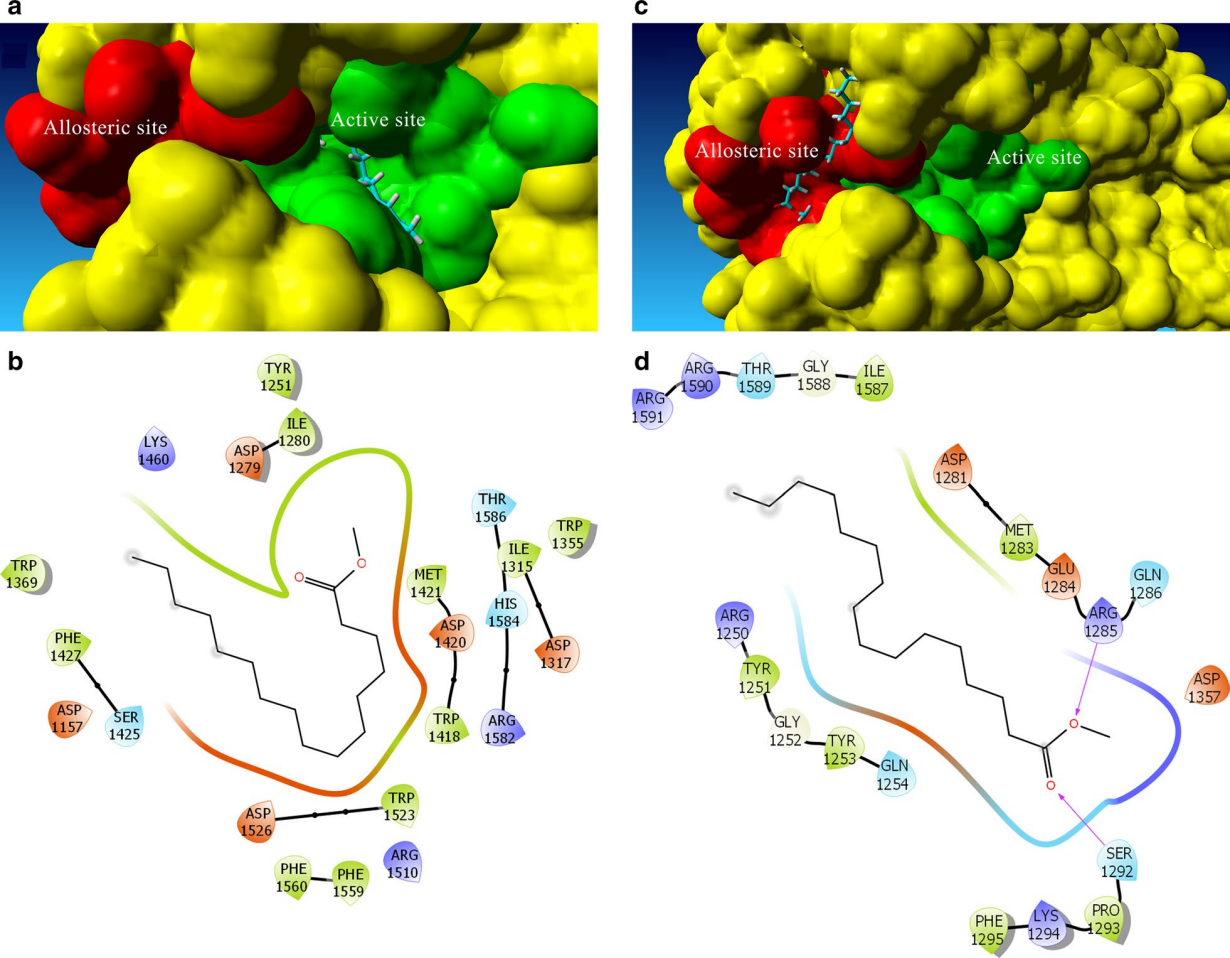
Predicted poses of methyl palmitate within the **a** active and **b** allosteric sites of α-glucosidase. 2D diagrams of enzyme-inhibitor complexes in **c** active and **d** allosteric site

Experimental results showed that methyl palmitate and tripalmitin acted as mixed inhibitors. Two different docking studies were carried out: one in the active site and the second one in a close allosteric site [18] which included Acarbose as a “cofactor” to block the entrance to the active site. Results from the docking study are shown in Figs. 6, 7 and Table 2. Rerank scores are calculated in Molegro Virtual Docker as an estimate of ligand binding, where lower values are associated with higher affinity.

**Table 2:** Results of molecular docking studies (Rerank score, more negative values indicate better theoretical affinity)

Ligand	Active site	Allosteric site
Methyl oleate	− 94.2873	− 84.4339
Methyl stearate	− 85.5979	− 79.7206
Methyl palmitate	− 80.5525	− 78.3820
FTG	− 133.662	^a^
Tripalmitin	− 94.8291	^a^

^a^These compounds occupy both sites simultaneously

**Fig. 8:**
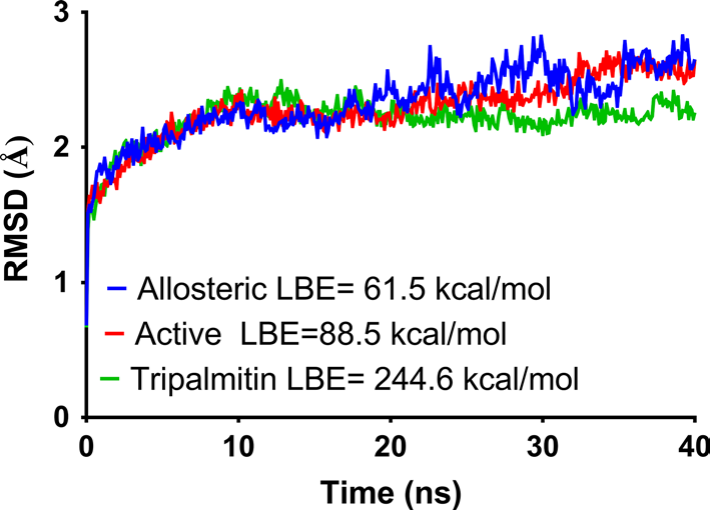
RMSD variations along MD simulation of a-glucosides complexes of tripalmitin and methyl palmitate in the active site and methyl palmitate in allosteric site. Ligand binding energies calculated by MM-PBSA are also included

Molecular dynamics (MD) simulations and molecular mechanics with Poisson–Boltzmann and surface area solvation (MM-PBSA) calculations were also performed to have a better understanding of the interactions found in the molecular modeling analysis. The results obtained are shown in Fig. 8.

### Artemia salina bioassay

Lethality assay using *Artemia salina* is a fast and cheap bioassay for assessing natural organic extracts biological activity which correlates well with cytotoxic activity. The LC_50_ value of *Artemia salina* obtained for the crude extract and active fractions (M and L) was higher than 1000, indicating an absence of cytotoxicity in these samples.

## Discussion

Microorganisms tend to use different strategies to combat adverse conditions within the ecosystem in which they develop, one of them being the production of metabolites [19]. In many cases, the specific function of each compound produced remains unknown, although it is possible to infer it based on the type of compound structure and function described in other areas. These correlations can be very useful to find potential applications of many of the compounds obtained from microorganisms. Triglycerides are the main form of energy storage in living organisms. In animals, most cells store small amounts of triglycerides, which appear as scattered droplets in the cytoplasm. This kind of metabolites are a reservoir of energy and carry out thermal insulation and protection functions [20]. The presence of high amounts of triglycerides in *Zasmidium* sp. suggests that these compounds could be performing functions similar to those mentioned above in this fungus because its host (the mangrove) lives in an ecosystem with great adverse conditions as mangroves such as extreme solar radiation during daytime.

In our understanding, this is the first report about the α-glucosidase inhibitory effect of triglyceride-type compounds, in order to be sure of our results we also evaluated the activity of tripalmitin and triolein standards purchased from Sigma-Aldrich. Both standards exhibited inhibitory activity of the enzyme α-glucosidase (IC50: 3.20 and 1.52 μM, respectively). We also evaluated the activity of methyl palmitate and glycerol (both obtained from methanolysis reaction) to get more information about the biological activity of tripalmitin, and we found that methyl palmitate inhibited α-glucosidase enzyme, while glycerol was inactive. Finally, the activity of oleic and stearic acids, constituent units of the triglycerides present in Fraction M, were also tested and both showed inhibitory activity with IC_50_ values of 0.23 and 0.81 µM. All these activity results corroborate the inhibitory effect of the α-glucosidase enzyme by triglyceride-type compounds, in addition to pointing out that the fatty acid units constituting the triglyceride are responsible for this activity.

Lineweaver–Burks plots showed that tripalmitin and methyl palmitate act as mixed inhibitors against α-glucosidase, suggesting that tripalmitin and methyl palmitate could bind to the free enzyme or the enzyme–substrate complex. When we analyze the obtained *K*_m_ values, we find that it decreases when the inhibitor binds to the enzyme–substrate complex, which in this case denotes an increase in the apparent affinity of the enzyme for the substrate.

Taking into account this evidences, molecular docking was used to predict binding conformations between inhibitors (tripalmitin and methyl palmitate) and the α-glucosidase enzyme, which could generate information for understanding the mechanism of action that could be used as the basis for the design of new inhibitors.

Rerank scores agree with previous reports that state that potency of inhibition is roughly correlated with the number of unsaturations [21–23] as fungal triglycerides (FTG) and methyl oleate had the lowest Rerank scores. Due to their size, both triglycerides, FTG, and tripalmitin can bind to both allosteric and active sites (Fig. 6a, b). FTG has a lower Rerank score than tripalmitin which coincides with experimental data. Analysis of the interaction of tripalmitin with specific residues of α-glucosidase reveals that hydrophobic interactions have an essential contribution to ligand binding as expected. However, hydrogen bond interactions of the carbonyl groups of the triglyceride with polar residues located inside the active site cavity (Gln1372 and Arg1377) seems also crucial for ligand binding.

Results from docking studies (Table 2) allow us to propose that while FAMEs prefer to bind to the active site, it is possible that they could bind, with slightly lower affinity, to the allosteric site even when the active site is occupied, explaining the mixed-inhibition behavior. Figure 7 shows the binding of methyl ester to the active site (Fig. 7a, b) and the allosteric site (Fig. 7c, d). Analysis of the interaction patterns reveals that, as excepted, most interactions are of hydrophobic nature. The presence of a hydrogen bonding group seems important for enzyme binding in both active and allosteric sites. This aspect would be necessary to take in consideration in the design of new inhibitors based on FAME template.

Even though the results of the docking study correlate with the results found in our experiments, it is a well-known fact that the efficiency of a docking study may decrease as the number of rotatable bonds increases [24]. Thus, we carried out MD simulations and MM-PBSA calculations, which are considered more rigorous than molecular docking [25]. Analysis of variations of Root-Median Standard Deviation (RMSD) along time (Fig. 8) suggests that the three complexes predicted from molecular docking are stable since RMSD fluctuations did not surpass 3 Å along all simulation time. In other hand, ligand binding energy (LBE) calculated using MM-PBSA shows that methyl palmitate has a slightly better affinity to the active site than to the allosteric site which coincides with docking scores. LBE calculated for tripalmitin complex suggest that it could have better binding than methyl palmitate, a fact that does not precisely correlate with experimental evidence from inhibition kinetics. The high flexibility of tripalmitin could be detrimental for binding to the active or allosteric site, and this could explain the lack of correlation with experimental results.

## Conclusions

Bioassay-guided fractionation of active extract from *Zasmidium* sp. strain EM5-10 against α-glucosidase allowed us to isolate one triglyceride and to detect a complex triglycerides mixture from a second active fraction. The structure of the active compound was established by spectroscopic analyses and comparison of the obtained data with those of the literature.

Tripalmitin (3.67 µM) and fungal triglycerides mixture (0.95 µg/mL) showed better inhibitory activity than Acarbose (positive control, IC_50_ 217.71 µM). To our knowledge, this is the first report on α-glucosidase inhibitory activity of triglycerides (tripalmitin and triolein, in our case). On the other hand, this type of compounds exhibited a mixed type of inhibition against *S. cerevisiae* α-glucosidase. Results from docking studies reveals that hydrophobic interactions have an essential contribution to ligand binding as expected. However, hydrogen bond interactions of the carbonyl groups of the tripalmitin with polar residues located inside the active site cavity (Gln1372 and Arg1377) seems also crucial for ligand binding.

Therefore, this fungus represents a new source of α-glucosidase inhibitors, which could possess beneficial properties for human health.

## Methods

### General experimental procedures

NMR spectra were acquired on Jeol Eclipse 400 MHz. One-dimensional spectra were referenced to δ_H_ 7.26, δ_C_ 77.0 for CDCl_3_ (used solvent). ESI mass spectra were recorded on a micrOTOF-Q II spectrometer, Bruker Daltonics, Germany. The purification of the compounds was carried out on SPE cartridge on C18 (6 mL/1000 mg, Macherey–Nagel, Düren, Germany). Purification solvents were HPLC grade and used without further purification. Methanolysis chemicals were reagent grade.

### Fungal isolation

The endophytic fungus isolate reported in this study was isolated from mature leaves of *L. racemosa* (Combretaceae), collected in Juan Diaz, Panama, in 2012. It was isolated in Bacto™ Malt Extract agar with 1% artificial sea salt (MEA) and 0.02% antibiotic, using a standard protocol. Briefly, leaves were surface-sterilized, first they were immersed in 70% ethanol solution, followed by 1% sodium hypochlorite solution, then in 70% ethanol solution, and finally leaves were washed with sterile distilled water. Each immersion process was 2 min. Leaves were cut into several 1 mm^2^ pieces and placed in individual Petri dishes with Bacto™ Malt Extract agar with 1% artificial sea salt (MEA) and Chloramphenicol as antibiotic. Petri dishes were checked daily to observe the growth of fungi, which were isolated and transferred to new Petri dishes for purification.

### DNA sequence identification

Identification of the endophytic fungus isolate was made by comparison of the nuclear ribosomal internal transcribed spacer region (ITS) nucleotide sequence of the fungus with those in the National Institutes of Health genetic sequence database (GenBank), using the Basic Local Alignment Search Tool (BLAST) [26]. DNA sequence identity equal or above 99% for the entire ITS region (ITS 1, the 5.8S gene, and ITS2) with type culture sequence and evaluation of taxonomic literature [15] was considered as the criterion for assigning genus name to the isolated fungus. Extraction, PCR and sequencing of DNA were done as reported in Mejia et al. [27].

### Small-scale culture

For screening of the initial activity against α-glucosidase enzyme, the fungus was grown in small-scale using 10 Petri dishes with MEA inoculated with small pieces actively growing mycelium (0.5 cm^2^). The cultures were incubated for 21 days at room temperature.

### Large-scale culture

For chemical studies, *Zasmidium* sp. strain EM5-10 was grown in 500 Petri dishes, each containing MEA, dishes were individually inoculated with a 1 cm^2^ agar plug taken from a stock culture with the fungus under study. The cultures were incubated for 21 days at room temperature.

### Production of extracts

After the incubation period, the Petri dishes were freeze-dried for 48 h. Mycelia and MEA were extracted with ethyl acetate. The solvent was evaporated, and extract concentrated under reduced pressure to a semisolid paste using a Buchi rotary Evaporator (R-215) to obtain 1.5 g of crude extract.

### Isolation of compounds

The organic extract was fractionated by SPE cartridge on C18 (6 mL/1000 mg). The SPE cartridge was first conditioned with methanol (12 mL) and then equilibrated by water (12 mL). The crude extract (1.5 g) was dissolved with 6 mL of 10% MeOH and sample was loaded onto the SPE column. The column was eluted with a gradient of water:MeOH (9:1 → 0:1). Altogether, 15 fractions (12 ml each) were collected as primary fractions (FA to FP). Evaluation of all fractions showed that only fractions L and M exhibited activity (97% and 96% of α-glucosidase inhibition, respectively) at a concentration of 6.25 µg/mL.

### Methanolysis

Tripalmitin was subjected to methanolysis using Kato et al. and Ichibara et al. protocols, with small modifications [28, 29]. Briefly, to 50 mg of sample diluted in 1 mL of ethyl ether was added 9 ml of 0.7 M KOH in MeOH and 1 mL of MeOH. The reaction mixture was kept overnight at room temperature without agitation. Next day, pH of the reaction mixture was adjusted to 3.0 with 0.5 N H_2_SO_4_, distilled water was added to obtain solvent phases. The reaction was monitored by thin layer chromatography using as a mobile phase hexane/acetone/acetic acid (95:5:0.5 v/v). Spots were visualized using sulphuric acid solution and heating the TLC to 135 °C [30]. FAMEs were recovered washing the mixture solution three times with hexane. Organic extraction was concentrated under reduced pressure using a Buchi rotary Evaporator (R-215).

### α-Glucosidase inhibitory assay

The α-glucosidase inhibitory assay was performed according to the protocol established in our laboratory [31]. α-Glucosidase from baker’s yeast purchased from Sigma Chemical Co. The inhibition was measured spectrophotometrically at pH 7.0 and 37 °C employing 2 mM p-nitrophenyl α-d-glucopyranoside (PNP-G) as a substrate and 32 mU/mL of the enzyme, in 100 mM potassium phosphate buffer (enzyme stock). Acarbose was dissolved in phosphate buffer, and serial dilutions (in order to obtain the IC_50_) were prepared and employed as positive control. The absorbance (A) of 4-nitrophenol released by the hydrolysis of PNP-G was measured at 400 nm by Synergy HT Bio Tek microplate spectrophotometer. A 20 µL of Acarbose or test compounds solution was incubated for 7 min with 150 µL of enzyme stock at 37 °C. After incubating, 150 µL of the substrate was added and further incubated for 20 min at 37 °C. All assays are performed in 96-well microplates (Greiner bio-one 655101) in duplicate. The activity of samples was calculated as a percentage in comparison to a control (DMSO or MeOH instead of sample solution) according to the following equation:

$$\% Inhibition = \left( {{\raise0.7ex\hbox{${\left( {\Delta A_{control } - \Delta A_{sample} } \right)}$} \!\mathord{\left/ {\vphantom {{\left( {\Delta A_{control } - \Delta A_{sample} } \right)} {\Delta A_{control} }}}\right.\kern-0pt} \!\lower0.7ex\hbox{${\Delta A_{control} }$}}} \right) \times 100\%$$

The concentration required to inhibit activity of the enzyme by 50% (IC_50_) was calculated by regression analysis [32].

### Standard reagents

Tripalmitin (glyceryl tripalmitate, purity ≥ 99%, T5888), triolein (glyceryl trioleate, purity ≥ 99%, T7140), glycerol (purity ≥ 99%, G6279), methyl stearate (purity ≥ 96%, W504807) and methyl oleate (purity ≥ 99%, 311111) were purchased from Sigma Chemical Co. (St. Louis, MO).

### Docking study

Fungal triglyceride and methyl esters of fatty acids were constructed in Spartan’10 [33], and its geometry was optimized using MMFF force field. Protein–ligand docking studies were carried out in C-terminal domain of human intestinal α-glucosidase (Accession Code: 3TOP) [34] which was retrieved from the Protein Data Bank. [35]. Molecular docking calculations were performed using Molegro Virtual Docker v. 6.0.1. [36]. Before docking, all of the solvent and co-crystallized ligand molecules were removed. As experimental data showed that fungal triglyceride exhibits mixed inhibition of α-glucosidase, some considerations were taken. A search for potential cavities was carried out finding five potential binding sites, one corresponding to the active site. Among the other four cavities, the one that was closer to active site was selected as the potential binding site of a non-competitive inhibitor; this approach has been previously reported [18]. As these cavities were close enough, they were merged in one bigger cavity, to analyze if the studied compounds preferred the active or allosteric site. A sphere of 18 Å radius was centered in the merged cavity for searching. Assignments of the charges and protonation states were based on standard templates as part of the program. Flexible ligand models and MolDock Optimizer algorithm were used. Orientations of the ligands into the cavity were searched and ranked based on their scores. The RMSD threshold for multiple cluster poses was set to < 1.00 Å. The docking algorithm was set to 10,000 maximum iterations with a simplex evolution population size of 250 and a minimum of 100 runs for each ligand. Poses with the lowest Rerank scores were selected for further analysis. Acarbose was also docked into the cavity to assess the efficacy of this procedure. The RMSD of the pose of the lowest Rerank score was calculated. RMSD was lower than 2 Å, indicating that the methodology used in the molecular docking studies is appropriate. An additional docking study was carried out maintaining the Acarbose in the active site, to analyze if the studied compounds could bind to the allosteric site when a substrate is present. The same methodology described above was carried out, except that for blocking access to active site, Acarbose was kept in it.

### Molecular dynamics simulations

The docking poses of tripalmitin (occupying both allosteric and active sites as shown in Fig. 6) and methyl palmitate (one pose with palmitate occupying the active site and another one occupying the allosteric site as shown in Fig. 7) with α-glucosidase were further analysed using molecular dynamics (MD) to analyse the stability and conformational changes of the predicted complexes. The MD simulations were performed using YASARA Dynamics v.18.4.24 [37] using the AMBER 14 force field [38]. The initial structures were taken from the poses with the lowest docking score of each complex and were placed in a cell box that had an extension of 10 Å larger on each side of the protein and was filled with water molecules. Periodic boundary conditions (PBC) were applied. The temperature was set at 298 K, water density to 0.997 g/cm^3^ and pH to a value of 7.4. Sodium (Na^+^) and chlorine (Cl^−^) ions were included to provide conditions that simulate a physiological solution (NaCl 0.9%). Particle Mesh Ewald algorithm was applied to evaluate long-range electrostatic interactions, the cut-off for van der Waals interactions was set to 8 Å. A multiple step of 2.5 fs was set, data were collected per 100 ps to a final simulation time of 40 ns. Results were analysed with a script included as part of YASARA software and included Root Mean Square Deviation (RMSD), Root Mean Square Fluctuations (RMSF) and ligand binding energy calculations using MM-PBSA.

### Brine shrimp lethality assay

In vitro lethality assay of *Artemia salina* was used for detecting toxicity from the crude fungal extract and their fractions [39]. Brine shrimp eggs were placed in seawater (3.8% w/v sea salt in distilled water) and incubated at 28 °C. Eggs were hatched within 48 h providing a large number of larvae (nauplii). Serial dilutions (1000, 500, 250 and 125 ppm) were made in separated wells of 96-well microplate. Nauplii were placed in each well by pipetting them until deposited 10–15 organism. Each concentration was assessed by triplicate. The percentage lethality was determined by comparing the mean surviving larvae of the test and control wells. Lethal concentrations values were obtained from the best-fit line plotted concentration versus percentage lethality. Potassium dichromate was used as a positive control in the bioassay while the negative controls were wells that contain only the solvent used for the preparation of the test samples.

## References

[1] Spalding M, Kainuma M, Collins L (2010). World atlas of mangroves.

[2] Patra JK, Mohanta YK (2014). Antimicrobial compounds from mangrove plants: a pharmaceutical prospective. Chin J Integr Med.

[3] Sithranga Boopathy N, Kathiresan K (2010). Anticancer drugs from marine flora: an overview. J Oncol.

[4] Zhou Z, Guo Y (2012). Bioactive natural products from Chinese marine Flora and Fauna. Acta Pharmacol Sin.

[5] Debbab A, Aly AH, Proksch P (2013). Mangrove derived fungal endophytes—a chemical and biological perception. Fungal Divers.

[6] Wang K-W, Wang S-W, Wu B, Wei J-G (2014). Bioactive natural compounds from the mangrove endophytic fungi. Mini Rev Med Chem.

[7] Bischoff H (1994). Pharmacology of glucosidase inhibitor. Eur J Clin Invest.

[8] Huang HB, Feng XJ, Liu L, Chen B, Lu YJ, Ma L, She ZG, Lin YC (2010). Three dimeric naphtho-γ-pyrones from the mangrove endophytic fungus *Aspergillus tubingensis* isolated from *Pongamia pinnata*. Planta Med.

[9] Huang H, Feng X, Xiao Z, Liu L, Li H, Ma L, Lu Y, Ju J, She Z, Lin Y (2011). Azaphilones and p-Terphenyls from the mangrove endophytic fungus *Penicillium chermesinum* (ZH4-E2) isolated from the South China Sea. J Nat Prod.

[10] Song Y, Wang J, Huang H, Ma L, Wang J, Gu Y, Liu L, Lin Y (2012). Four eremophilane sesquiterpenes from the mangrove endophytic fungus *Xylaria* sp. BL321. Mar Drugs.

[11] Liu Y, Yang Q, Xia G, Huang H, Li H, Ma L, Lu Y, He L, Xia X, She Z (2015). Polyketides with α-glucosidase inhibitory activity from a mangrove endophytic fungus, *Penicillium* sp. HN29-3B1. J Nat Prod.

[12] Chen S, Liu Y, Liu Z, Cai R, Lu Y, Huang X, She Z (2016). Isocoumarins and benzofurans from the mangrove endophytic fungus *Talaromyces amestolkiae* possess α-glucosidase inhibitory and antibacterial activities. RSC Adv.

[13] Cui H, Liu Y, Nie Y, Liu Z, Chen S, Zhang Z, Lu Y, He L, Huang X, She Z (2016). Polyketides from the mangrove-derived endophytic fungus *Nectria* sp. HN001 and their α-glucosidase inhibitory activity. Mar Drugs.

[14] Lopez D, Cherigo L, de Sedas A, Spadafora C, Martínez-Luis S (2018). Evaluation of antiparasitic, anticancer, antimicrobial and hypoglycemic properties of organic extracts from Panamanian mangrove plants. Asian Pac J Trop Med.

[15] Braun U, Crous PW, Schubert K, Shin HD (2010). Some reallocations of *Stenella* species in *Zasmidium*. Schlechtendalia.

[16] Vlahov G (1999). Application of NMR to the study of olive oils. Prog Nucl Magn Reson Spectrosc.

[17] Simova S, Ivanova G, Spassov SL (2003). Alternative NMR method for quantitative determination of acyl positional distribution in triacylglycerols and related compounds. Chem Phys Lipids.

[18] Javaid K, Saad SM, Rasheed S, Moin ST, Syed N, Fatima I, Salar U, Khan KM, Perveen S, Choudhary MI (2015). 2-Arylquinazolin-4(3H)-ones: a new class of α-glucosidase inhibitors. Bioorg Med Chem.

[19] Paul VJ, Arthur KE, Ritson-Williams R, Ross C, Sharp K (2007). Chemical defenses: from compounds to communities. Biol Bull.

[20] Alvarez HM, Steinbüchel A (2002). Triacylglycerols in prokaryotic microorganisms. Appl Microbiol Biotechnol.

[21] Miyazawa M, Yagi N, Taguchi K (2005). Inhibitory compounds of α-glucosidase activity from *Arctium lappa* L. J Oleo Sci.

[22] Nguyen TH, Kim SM (2015). α-glucosidase inhibitory activities of fatty acids purified from the internal organ of sea cucumber *Stichopus japonicas*. J Food Sci.

[23] Liu B, Kongstad KT, Wiese S, Jäger AK, Staerk D (2016). Edible seaweed as future functional food: identification of α-glucosidase inhibitors by combined use of high-resolution α-glucosidase inhibition profiling and HPLC–HRMS–SPE–NMR. Food Chem.

[24] Erickson JA, Jalaie M, Robertson DH, Lewis RA, Vieth M (2004). Lessons in molecular recognition: the effects of ligand and protein flexibility on molecular docking accuracy. J Med Chem.

[25] Mobley DL, Dill KA (2009). Binding of small-molecule ligands to proteins: “what you see” is not always “what you get”. Structure.

[26] Altschul SF, Gish W, Miller W, Myers EW, Lipman DJ (1990). Basic local alignment search tool. J Mol Biol.

[27] Mejía LC, Castlebury LA, Rossman AY, Sogonov MV, White JF (2011). A systematic account of the genus *Plagiostoma* (Gnomoniaceae, Diaporthales) based on morphology, host-associations, and a four-gene phylogeny. Stud Mycol.

[28] Kato A, Ando K, Kodama K, Tamura G, Arima K (1969). Identification and chemical properties of antitumor active monoglycerides from fungal mycelia. Studies on antiviral and antitumor antibiotics. X. J Antibiot.

[29] Ichihara K, Yamaguchi C, Araya Y, Sakamoto A, Yoneda K (2010). Preparation of fatty acid methyl esters by selective methanolysis of polar glycerolipids. Lipids.

[30] Ichihara K, Shibahara A, Yamamoto K, Nakayama T (1996). An improved method for rapid analysis of the fatty acids of glycerolipids. Lipids.

[31] López D, Cherigo L, Spadafora C, Loza-Mejía MA, Martínez-Luis S (2015). Phytochemical composition, antiparasitic and α-glucosidase inhibition activities from *Pelliciera rhizophorae*. Chem Cent J.

[32] Copeland RA (2000). Enzymes: a practical introduction to structure, mechanisms and data analysis.

[33] Spartan’10 for Windows. Wavefunction Inc., Irvine, CA, USA

[34] Ren L, Qin X, Cao X, Wang L, Bai F, Bai G, Shen Y (2011). Structural insight into substrate specificity of human intestinal maltase-glucoamylase. Protein Cell.

[35] Berman HM, Westbrook J, Feng Z, Gilliland G, Bhat TN, Weissig H, Shindyalov IN, Bourne PE (2000). The protein data bank. Nucleic Acids Res.

[36] Thomsen R, Christensen MH (2006). MolDock: a new technique for high-accuracy molecular docking. J Med Chem.

[37] Krieger E, Vriend G (2015). New ways to boost molecular dynamics simulations. J Comput Chem.

[38] Maier JA, Martinez C, Kasavajhala K, Wickstrom L, Hauser KE, Simmerling C (2015). ff14SB: improving the accuracy of protein side chain and backbone parameters from ff99SB. J Chem Theory Comput.

[39] Meyer BN, Ferrigni NR, Putnam JE, Jacobsen LB, Nichols DE, McLaughlin JL (1982). Brine shrimp: a convenient general bioassay for active plant constituents. Planta Med.

